# Evaluation of the national surveillance of Legionnaires' disease in Norway, 2008-2017

**DOI:** 10.1186/s12889-019-7981-9

**Published:** 2019-12-03

**Authors:** Cecilia Wolff, Heidi Lange, Siri Feruglio, Line Vold, Emily MacDonald

**Affiliations:** 1 Division for Environmental Health and Infectious Disease Control, Norwegian Institute of Public Health, Postboks 222 Skøyen, 0213 Oslo, Norway; 2 European Programme for Intervention Epidemiology Training (EPIET), European Centre for Disease Prevention and Control (ECDC), Stockholm, Sweden

**Keywords:** Legionnaires’ disease, Legionellosis, Surveillance evaluation, Disease outbreak, Travel-associated, Public health

## Abstract

**Background:**

In Norway, Legionnaires’ disease is reportable upon clinical suspicion to public health authorities and mandatorily notifiable through the Norwegian surveillance system for communicable diseases (MSIS) for both clinicians and laboratories. In the summer of 2017, several European countries reported high notification rates for Legionnaires’ disease, which was not observed in Norway. We evaluated MSIS to assess if it meets its objectives of detecting cases and trends in incidence of Legionnaires’ disease.

**Methods:**

We retrieved MSIS data from 2008 to 2017 and calculated timeliness as days from sampling to notification, and internal completeness for key variables as the proportion of observations with a value. Where possible, we assessed internal validity on the presence of a plausible value. To estimate external completeness and validity we linked MSIS with hospital reimbursement claims in the Norwegian Patient Registry. To assess acceptability and representativeness, we surveyed doctors in 39 hospitals on their units’ diagnostic and notification procedures, and their use of MSIS.

**Results:**

There were 438 notified cases. Internal completeness and internal validity were high for key variables (≥95%). The median delay from sampling to notification was 4 days.

There were 73 patients in MSIS only, 70 in the Norwegian Patient Registry only, and 351 in both registers. The external completeness of MSIS was 83% (95% CI 80–86%). For external validity, the positive predictive value of MSIS was 83% (95% CI 79–86%).

Forty-seven respondents from 28 hospitals described testing procedures. These were inconsistent: 29 (62%) reported no systematic application of criteria for requesting legionella testing. Eighteen (38%) reported testing all patients with suspected pneumonia and a travel history. Thirty-one (66%) found the notification criteria clear.

**Conclusions:**

Our results suggest that the surveillance in MSIS can detect incidence changes for Legionnaires’ disease over time, by place and person, but likely does not detect every case diagnosed in Norway.

We recommend wider investigation of diagnostic procedures in order to improve representativeness and awareness of MSIS notification criteria among clinicians in order to improve acceptability of the surveillance. We also recommend a more comprehensive assessment of whether patients only registered in the Norwegian Patient Registry were true Legionnaires’ disease cases.

## Background

Legionnaires’ disease (LD) is an atypical pneumonia caused by the *Legionella* bacterium. The majority of human infections are caused by *Legionella pneumophila,* mainly serogroups 1 and 6 (1). Other species such as *L. longbeachae* can also cause disease (2). Although LD is generally an uncommon and sporadic infection with a low attack rate, case fatality rate is high, typically 10% in Europe and between 15 and 34% for nosocomial cases (3). The incubation period for LD is 2–10 days with a median of 6–7 days (3). Worldwide, 75–80% of the reported cases are over 50 years and 60–70% are male (4).

*Legionella* bacteria are ubiquitous in nature. The *pneumophila* serotype is primarily found in man-made freshwater reservoirs, especially standing water where biofilms can develop (5, 6). Legionella bacteria are also found elsewhere in the environment. For example, *L. longbeachae* is often found in soil, compost, and potting-mixes (2, 7). The main infection route for *Legionella* is through inhalation of contaminated aerosols. Outbreaks of LD in Norway have been linked to cooling towers, hot tubs, and an industrial air scrubber (8).

A urine antigen test (UAG) has become the most commonly used diagnostic method in Norway, as in most other countries (3, 9). This test only detects *Legionella pneumophila* serogroup 1 with an overall (pooled, weighted) sensitivity of 74% (from 54 to 91% depending on brand) and overall specificity of 99% (10). The sensitivity is higher in community acquired and travel-associated cases compared to nosocomial cases (11). Sensitivity is also higher in severe clinical illness and when urine is analysed after concentration methods (12). Ideally, a positive UAG result should be confirmed by culture and isolation. The reference standard for diagnosis of LD is culture and isolation from bronchoalveolar lavage (BAL), sputum or biopsy or fine needle aspiration from lung tissue (3). Nucleic acid detection in BAL, sputum or lung tissue is also carried out by hospital laboratories in Norway. Diagnosis by serology is another alternative, although seroconversion in most culture-positive patients is not detectable until at least 3 weeks after infection, and never detectable in up to 25% of culture positive patients (1). Challenges with LD surveillance include under-diagnosis due to the UAG only detecting one serogroup of *L. pneumophila* (10). If no other test is applied, LD caused by other species or serogroups may go undetected. If the patient is successfully treated, LD may never become established. This is also true for patients not tested for legionella at all, which could include those with less severe clinical presentation. If notification for surveillance is carried out by laboratories, bedside testing, which is possible with UAG, may reduce the notification rate.

In Norway, LD has been mandatorily notifiable to the Norwegian Surveillance System for Communicable Diseases (MSIS) since 1980. The majority of patients are infected abroad, but major local outbreaks of LD occurred in 2001 (13) and 2005 (14). In the 2005 outbreak, 10 out of 56 registered patients died (14). Before 2001, the annual number of reported cases was fewer than five cases most years. After the two large outbreaks, awareness increased. From 2006 to 2017, the average annual notified incidence was 42 cases, with an increasing trend.

In the US, a marked increase in incidence of notified cases was seen from year 2003 (15), and a three-fold increase from year 2000 to 2009 (16). In the EU/EES area, the age-standardised incidence rate of notified cases increased from 0.97 cases/100000 in 2011 (17) to 1.8 per 100,000 in 2017, which was a 30% increase compared to 2016 (18). Notified LD incidence normally peaks during the summer months. This was reflected in the summer months of 2017, when the EU/EES experienced the highest notification rate in five years. However, there was a decrease in notified cases in Norway during the same time period (18, 19), but no known interventions that could explain a decrease in LD incidence. This difference in Norway raised concerns of under-reporting to MSIS, and warranted an evaluation of the national LD surveillance. We carried out an evaluation of the surveillance system to determine whether it accurately detects cases and outbreaks and describes trends, in order to be able to give recommendations for improvement of the surveillance.

## Methods

We applied the guidelines for evaluation of surveillance systems given by the European Centre for Disease Prevention and Control (ECDC) (20) and the U.S. Centers for Disease Control and Prevention (CDC) (21) to evaluate the MSIS system attributes data quality (internal completeness and internal validity), timeliness, representativeness, acceptability, external completeness, and external validity.

### Description of the surveillance system

#### Objectives of MSIS

The overall objective of infectious disease surveillance through MSIS, which is common to all 72 notifiable diseases, is to contribute to the surveillance of communicable diseases in people in Norway through continuous and systematic collection, analysis, interpretation, and reporting of data on incidence of communicable diseases. The two specific objectives of MSIS that we evaluated with regard to LD are to describe disease incidence over time, by geographic and demographic parameters, and, to detect and enable investigation of outbreaks of infectious diseases, which for some diseases including LD means detection also of single cases (22).

#### Case definition

The MSIS case definition for LD is:” *Pneumonia and laboratory confirmation of Legionella spp. in airway secretions, lung tissue, or blood by isolation or nucleic acid detection, or Legionella spp. in urine, airway secretions, or lung tissue by antigen detection, or Legionella antibodies (seroconversion or significant increase in antibody titre in paired samples or a single sample with increased antibody titre)”.*

#### Data sources for LD cases

According to the MSIS legislation, all LD cases diagnosed in Norway are notifiable to MSIS regardless of the country of residence (22). The Norwegian Institute of Public Health (NIPH) is responsible for the collection and management of data in MSIS (22). The data providers for LD are clinicians (in primary care, hospitals, and other health care institutions) and microbiological laboratories. All are required to notify new cases on the day of diagnosis (22). Clinicians notify by using a standardized form, which includes variables on patient demographics, clinical presentation, disease transmission and laboratory findings. The national reference laboratory at NIPH or at Stavanger University Hospital receive cultures of *Legionella* spp. for confirmation, typing and biobanking.

In addition, clinicians and other health personnel are required to immediately report suspected LD to the municipality medical doctor (MMD) or the NIPH (22), while an investigation is started to identify the source of infection. If the disease is travel-related, NIPH notifies the European Legionnaires Disease Surveillance Network (ELDSNet) of ECDC. NIPH staff can add the information from an immediate report to MSIS. When the MSIS notification(s) are received, the date of an immediate report is replaced by the notification date.

#### Data entry

Data is reported either electronically or paper based to MSIS. Notifications from laboratories and clinicians are linked using the patient’s personal identification number. The reporting clinician or laboratory receive reminders from MSIS after three weeks if there are missing or unclear variables. Variables like name, birth date, sex, residential address, and country of birth are validated by matching against the population registry. Other variables collected include diagnosis, date of symptom onset, date of sampling, probable date of infection, reason for testing (symptoms, routine or contact tracing), description of symptoms, hospitalization status, outcome of illness, and place of infection.

### Evaluation of the LD surveillance system attributes

#### Data quality (internal completeness and validity) and timeliness

To evaluate data quality (internal validity and internal completeness) and timeliness, we queried the MSIS database for all records with LD and date of illness onset from 1 January 2008 to 31 December 2017 (data retrieved in February 2018). The variables included key parameters such as dates, patient data, details on the geographical location for transmission and any association with travel, diagnostics, hospitalization, and who notified the case. Data from the MSIS database were summarized with the number of cases by time (year), place (residential county), and person (sex and age group). We calculated the number of cases notified by a clinician, a laboratory, or both.

We assessed each selected variable for internal completeness by calculating the number and proportion of records without unknown or missing values.

Internal validity refers to whether the value of a variable in the surveillance data is correct, for example if the date of illness onset was before or the same as test date or if tested specimen matched test methodology. For each assessed variable the number and proportion of records that were valid were calculated.

Timeliness refers to the time taken between different steps of the surveillance system as a proxy for measuring whether the system enables timely action. We calculated the median number of days between the reported date of illness onset, sampling for diagnostic test and notification. After removing the non-valid observations, we repeated the calculation.

#### External completeness and validity

To evaluate the attributes external completeness and external validity, we compared registrations in MSIS against the Norwegian Patient Registry (NPR). NPR registers all reimbursement claims from hospitals to the Directorate of Health (HDir). Patients with suspected (under investigation) or confirmed LD are registered with a specific ICD-10 code in NPR. If a suspected LD case is subsequently diagnosed with another condition, any already submitted reimbursement claim(s) with the LD diagnosis will not be retrospectively corrected in NPR.

The NPR data included sex and birth year for the patient, dates of admission and discharge, type of visit (hospitalization, day treatment, or outpatient), which hospital and hospital group, and all diagnostic codes (up to 20). A patient hospitalized for several days could have one or several claims for this event.

**Fig. 1:**
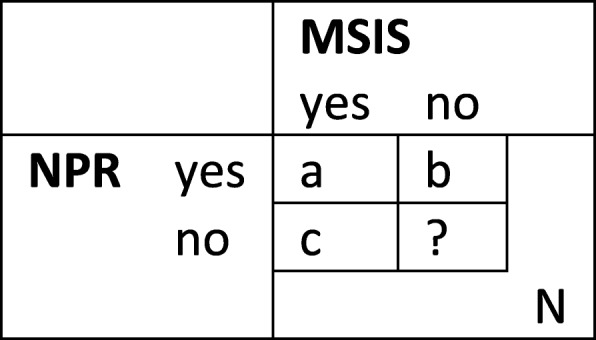
Two-by-two table for evaluation of external completeness and external validity of MSIS against NPR

Patients in MSIS and NPR from 2008 to 2017 were linked by personal identification number. We summarized the number and percentage of patients found in both NPR and MSIS, in NPR only, and in MSIS only, in total and by year, using the annotation in Fig. 1

.

Data in NPR are not collected for surveillance purposes, and cannot be used as a gold standard to evaluate MSIS against. To estimate the external completeness of MSIS we used methods for comparing two independent data sources.

As a first step, we used capture-recapture according to Chapman (20) to estimate the total number of patients (N) with LD, using the annotation in Fig. 1 with cases in both NPR and MSIS (a), cases only in NPR (b), and cases only in MSIS (c):

$$ \mathsf{N}=\left(\mathsf{a}+\mathsf{b}+\mathsf{1}\right)\ast \left(\mathsf{a}+\mathsf{c}+\mathsf{1}\right)/\left(\mathsf{a}+\mathsf{1}\right)-\mathsf{1} $$

External completeness refers to the ability of the system to capture diagnosed cases. Using the estimated total number of LD cases, we calculated the external completeness of MSIS as the sum of cases in MSIS and NPR (a) and cases only in MSIS (c) divided by the estimated total number of patients (N):

$$ {\mathsf{EC}}_{\mathsf{MSIS}}=\left(\mathsf{a}+\mathsf{c}\right)/\mathsf{N}\ \left(\mathsf{21}\right) $$

External validity refers to whether notified cases are true cases, and to assess this, we combined several methods (20). First, we calculated the concordance MSIS-NPR using the annotation in Fig. 1 with cases in both NPR and MSIS (a), cases only in NPR (b), and cases only in MSIS (c):

$$ \mathsf{Proportion}\ \mathsf{of}\ \mathsf{all}\ \mathsf{patients}\ \mathsf{in}\ \mathsf{concordance}=\mathsf{a}/\left(\mathsf{a}+\mathsf{b}+\mathsf{c}\right) $$

$$ \mathsf{Proportion}\ \mathsf{of}\ \mathsf{contradictory}\ \mathsf{patients}\ \mathsf{in}\ \mathsf{MSIS}=\mathsf{c}/\left(\mathsf{a}+\mathsf{c}\right) $$

$$ \mathsf{Proportion}\ \mathsf{of}\ \mathsf{contradictory}\ \mathsf{patients}\ \mathsf{in}\ \mathsf{NPR}=\mathsf{b}/\left(\mathsf{a}+\mathsf{b}\right) $$

Secondly, we calculated the positive predictive value (PPV) of MSIS as cases in both MSIS and NPR (a) divided by the sum of cases in both MSIS and NPR (a) and only in NPR (b):

$$ {\mathsf{PPV}}_{\mathsf{MSIS}}=\mathsf{a}/\left(\mathsf{a}+\mathsf{c}\right)\ \left(\mathsf{21}\right) $$

To identify if any hospitals contributed more to the under-reporting to MSIS than others, we calculated the proportion of each hospital’s patients in NPR that were only found in NPR.

To assess if there were any non-linked patients in NPR and MSIS, we manually compared the patients found in NPR only with those only found in MSIS by hospital, sex, test date (MSIS) with admission date (NPR), and year of birth (NPR) with age group (MSIS). If there was a match on all these variables, with test date and admission date within one month, we considered the patient a likely match.

#### Representativeness and acceptability

We developed a survey questionnaire (Additional file 1) and distributed it to all hospitals with any notified LD cases in years 2013 to 2017. We sent the survey to the official email address of 20 hospital groups, representing 39 hospitals. The email cover letter explained the purpose of the survey, its context as part of a larger evaluation, and included a link to the online questionnaire (23). We asked for the email to be forwarded to the chief medical doctor of units that treat LD patients, for dissemination among senior doctors within each unit. We distributed the email on 7 September 2018 with reminders to five non-responders on 2 October, and three on 8 October. The raw data were saved on 1 Nov.

A representative public health surveillance system accurately describes the occurrence of a health-related event over time and its distribution in the population by place and person. We assessed representativeness through questions regarding which patients are tested for *Legionella*, diagnostic test methods that are used, and routines for immediate reporting and notification of LD cases. Acceptability reflects the willingness of persons and organisations to participate in the surveillance system. We assessed acceptability through questions on the respondents’ knowledge of the mandatory notification, and their own use of MSIS incidence data. We described survey data with frequencies and percentages, and reviewed free text comments and identified main themes identified.

### Performance of the surveillance system

We combined all results to assess how useful the system is in terms of meeting its stated objectives.

Data analysis was carried out in R v3.5.1 (24). Proportions and exact 95% binomial confidence interval were calculated using function biconf in the Hmisc package (25).

## Results

### Data quality and timeliness

There were 438 cases in MSIS and the number of notified cases per year ranged from 26 to 61. The cases were predominately male. The most frequent age categories were 60–69 years old (*n* = 140), 50–59 years old (*n* = 114), and 70–70 years old (*n* = 72). The five counties with the highest number of cases in the study period are the counties with the largest population. Of the 438 cases, 361 (82%) were notified by both clinician and laboratory, 13 (3%) only by the clinician, 40 (9%) by the laboratory, and 23 (5%) were “immediate reports” where either a notification had not been received, or the record was not deleted after a suspected LD case was given another diagnosis. Of the 438 cases, 345 (79%) were diagnosed by UAG, 64 (15%) by nucleic acid detection, 15 (3%) by culture, 13 (3%) by serology, and one case had unknown diagnostic test method. Only one diagnostic test method can be recorded.

The internal completeness of variables was at least 95% for key variables (Table 1). The internal validity (Table 2) was 92% for date of illness onset and sampling date, and 99% for date of reporting and diagnostic test method. The overall median timeliness was 10 days from illness onset until notification, and 4 days from sample date to notification (Table 3). Timeliness of notification was better for observations where UAG was used. When other diagnostic methods were applied, timelines had a skewed distribution with the median number of days from illness onset to notification being approximately double compared to use of UAG.

**Table 1:** Data quality, internal completeness, Legionnaire’s disease cases notified in MSIS, 2008 to 2017, Norway

Variable	Variable not empty and not ‘unknown’			Variable not empty		
	Complete observations (n)	%	95% CI	Complete observations (n)	%	95% CI
Place and source of infection						
Domestic or travel-associated	420	95.9	93.6;97.5	438	100	
Place where the patient was infected (country, city, other)	420	95.9	93.6;97.5	425	97.0	95.0;98.4
Which country (Norway or other)	400 of 420	95.2	92.7;97.1	400 of 420	95.2	92.7;97.1
Which county (if in Norway)	147 of 155	94.8	90.1;97.7	147 of 155	94.8	90.1;97.7
Reason for/purpose of travel abroad (if travel-associated)	251 of 265	94.7	91.3;97.1	254 of 265	95.8	92.7;97.9
Mode of transmission	365	83.3	79.5;86.7	386	88.1	84.7;91.0
Occupational infection	324	74.0	69.6;78.0	340	77.6	73.4;81.4
The clinic and test						
Test carried out (e.g. urine antigen test, PCR, serology)	437	99.8	98.7;100	438	100	
Sampled specimen (e.g. urine, sputum, BAL)	436	99.5	98.3;99.9	436	99.5	98.4;100
Hospitalised	433	98.9	97.4;99.6	433	98.9	97.3;99.6
Name of hospital if hospitalised	424 of 430	98.6	97.0;99.5	424 of 430	98.6	97.0;99.5
Why sampled (clinical illness, screening)	428	97.7	95.8;98.9	428	97.7	95.8;98.9
Clinical diagnosis/ indication (e.g. pneumonia, sepsis)	427	97.5	95.6;98.7	427	97.5	95.6;98.7
Outcome of disease (e.g. recovered, still ill, mortis)	360	82.2	78.3;85.7	388	88.6	85.2;91.4
Analysed by reference laboratory	20	4.6	2.8;6.9	21	4.8	3.0;7.2
The notification						
Notified by (laboratory, physician, or both)	437	99.8	98.7;100	437	99.8	98.7;100
Reporting hospital (name)	395	90.2	87.0;92.8	395	90.2	87.0;92.8
Name of reporting person or hospital clinic	354	80.8	76.8;88.4	354	80.8	76.8;88.4
Reporting type of clinic (e.g. medical, lung, infection)	134	30.6	26.3;35.1	134	30.6	26.3;35.1
Reporting institution (name of hospital, clinic, town)	81	18.5	15.0;22.5	81	18.5	15.0;22.5

Internal completeness for variables in MSIS for notified cases with a date of illness onset in 2008 to 2017 (*n* = 438)

**Table 2:** Data quality, internal validity, Legionnaires’ disease cases notified in MSIS, 2008 to 2017, Norway

Variable	Criteria for valid value	Valid observations		
		n	%	95% CI
Dates				
Date illness onset	< sample date and < date of reporting	404	92.2	89.3;94.6
Date sample taken	> date of illness onset and < = date of reporting	404	92.2	89.3;94.6
Date of reporting	> date of illness onset and > = sample date	433	98.9	97.3;99.6
Diagnostic test and diagnosis				
Analysed specimen	BAL, blood, expectorate, induced sputum, airway secretions, urine, biopsy, tissue sample	427	97.5	95.6;98.7
Test method and analyses specimen match	Material urine if method antigen test, and not antigen test if not urine	435	99.3	98.0;99.9
Reported by	At least one of reporting institution, person, hospital stated	432	98.6	97.0;99.5
Name of laboratory that analysed sample	Not ‘other’, ‘unknown’ or empty field	393	89.7	86.5;92.4

Internal validity for variables in MSIS for notified cases with a date of illness onset in 2008 to 2017 (*n* = 438)

**Table 3:** Timeliness of reporting for Legionnaires’ disease cases notified in MSIS, 2008 to 2017, Norway

	All observations						Valid observations								
	All, *n* = 438		Urine antigen test, *n* = 345		Not urine antigen test, *n* = 93		All			Urine antigen test			Not urine antigen test		
Days from to	Median	p10;p90	Median	p10;p90	Median	p10;p90	Median	p10;p90	n	Median	p10;p90	n	Median	p10;p90	n
Illness onset to test sampling	5	1;12	5	1;10	7	0.2;32.2	6	3;14	404	5	3;10	321	7	3;37.8	83
Illness onset to notified	10	5;31	9	4;19	19	8.2;699	10	3;31	404	9	4;19	321	19	9;796	83
Test sampling to notified	4	1;16	3	1;11	9	4;680	4	1;16	433	3	1;11	324	9	4;680	91

p10: 10th percentile, p90: 90th percentile

Cases notified in MSIS and a date of illness onset in the period 2008 to 2017 (n = 438), Norway. “Test” refers to diagnostic test for *Legionella*

### External completeness and validity

After linkage with NPR, we found 351 patients in both MSIS and NPR, 70 only in NPR, and 73 only in MSIS. There was no clear trend in increasing or decreasing number (or %) of patients found in only one of the registers (Table 4). The capture-recapture estimated the total number of patients (N) in the study period to 510 (95% CI 501–518). The external completeness of MSIS EC_MSIS_ was 83% (95% CI 80–86%). For the external validity of MSIS, the reporting concordance between MSIS and NPR was 71% (95% CI 69–75%). There were 17% contradictory patients in MSIS (95% CI 14–21%), and 17% contradictory patients in NPR (95% CI 13–21%). The PPV_MSIS_ was 83% (95%CI 79–86%).

**Table 4:** Number of Legionnaires’ disease patients in NPR and notified to MSIS, 2008 to 2017, Norway

	Year																					
	2008		2009		2010		2011		2012		2013		2014		2015		2016		2017		total	
Register	n	%	n	%	n	%	n	%	n	%	n	%	n	%	n	%	n	%	n	%	n	%
NPR	11	21	1	3	7	13	8	17	4	13	8	17	7	13	6	9	10	19	8	14	70	14
MSIS	10	19	8	24	7	13	1	2	5	17	8	17	7	13	6	9	11	20	10	17	73	15
Both	31	60	24	73	41	75	37	80	21	70	31	66	39	74	53	82	33	61	41	69	351	71
Total	52		33		55		46		30		47		53		65		54		59		494	

*NPR* Norwegian Patient registry. Data were linked by personal identification number. Case patients in MSIS without such a number (not Norwegian residents) were excluded

Among patients only found in NPR, 34 (49%) were treated in five of the 36 hospitals reporting at least one LD patients in NPR. Two hospitals had two patients each with LD registered in NPR during the study period but did not have any cases notified in MSIS. Of the patients only found in NPR, 62 (89%) were recorded as hospitalized and eight (11%) as outpatients. Of the eight outpatients, two had another seemingly non-related diagnostic code recorded. The remaining six had only the diagnostic code for LD. In comparison, of the 351 patients found in both MSIS and NPR, 342 (97%) were hospitalized, one (0.3%) was a day treatment, and eight (2%) were outpatients.

For the manual match of patients only in NPR with those only in MSIS we included another eight patients in MSIS without a Norwegian personal identification number (likely non-residents). Six of these eight patients in MSIS were among the 70 patients only in NPR.

### Representativeness and acceptability

The final survey response rate was 47 responses, representing 19 of 20 hospital groups. The non-responding group had only one hospital. There were differences in use of standardized diagnostic procedure and confirmation of test results between the surveyed hospital and hospital units (Table 5). A majority (62%) of respondents answered that there is no internal procedure to determine which patients should be tested for *Legionella*. Eight (17%) stated they test all patients with the clinical diagnosis pneumonia, and 18 (38%) responded that all patients with pneumonia and history of travel are tested for *Legionella*. The first line diagnostic test was UAG, used by 44 (94%), and mainly performed in the Laboratory Department (*n* = 35, 80%) (Table 5). Thirty-nine (89%) stated they use additional diagnostic methods if clinical suspicion is high and UAG negative.

**Table 5:** Survey answers regarding diagnostic procedures for Legionnaires’ disease, Norway, 2018

Question	Answer categories	n	%
Does the hospital or hospital unit have an internal procedure or algorithm for which patients to test for *Legionella*?	Yes, one that all doctors should follow	14	30
	No, it is up to each responsible doctor	29	62
	No, other	1	2
	Don’t know	3	6
Do you test all patients with suspected pneumonia for *Legionella*?	Yes	8	17
	No	38	81
	Don’t know	1	2
Do you test all patients with suspected pneumonia and a travel history for *Legionella*?	Yes	18	38
	No	23	49
	Don’t know	6	13
Which diagnostic test(s) do you use at your unit to diagnose/confirm *Legionella*? Choose all that apply	Urine antigen test	44	94
	Culture and isolation	23	49
	PCR	31	66
	Serology	3	6
	Don’t know	3	6
	Other (free text)	1	2
Where is the urine antigen test normally carried out? (*n* = 44)	At the hospital unit	3	7
	By the hospital laboratory	35	80
	It varies, both at the unit and by the laboratory	4	9
	Don’t know	1	2
	Other (free text)	1	2
Do you try to confirm a positive urine antigen test with culture and isolation of BAL or sputum? (*n* = 44)	Yes, always	7	16
	Yes, usually	21	48
	Rarely	8	18
	No, never	0	0
	Don’t know	8	18
What is your routine if the clinical suspicion is LD but the urine antigen test is negative? (*n* = 44)	Sample for analysis with another method (culture, PCR, serology)	39	89
	Don’t know	2	5
	Other (free text)	3	7
Do you try to confirm a positive PCR result with culture and isolation of BAL or sputum? (*n* = 31)	Yes, always	5	16
	Yes, usually	9	29
	Rarely	10	32
	No, never	1	3
	Don’t know	6	19
Do you try to confirm a positive serology result with another diagnostic method? (*n* = 3)	Yes, always	0	0
	Yes, usually	0	0
	Rarely	1	33
	No, never	0	0
	Don’t know	2	67
Is a positive serology result confirmed with a new sample with regard to titre increase? (*n* = 3)	Yes, always	0	0
	Yes, usually	0	0
	Rarely	1	33
	No, never	0	0
	Don’t know	2	67

Answers (*n* = 47) from a survey to doctors at Norwegian hospitals that notified at least one case with Legionnaires’ disease (LD) from 2013 to 2017

In terms of notification of LD cases, 10 (21%) respondents answered that they have no established routine regarding immediate reporting to the MMD or NIPH upon clinical suspicion (Table 6). Of those who have a routine or did not know if they have a routine (*n* = 37, 79%), four (11%) said they only notify to MSIS and do not report to the MMD or NIPH directly. Sixteen (34%) did not know if the notification criteria were clear (Table 6).

**Table 6:** Survey results regarding notification and use of MSIS data for Legionnaires’ disease, Norway, 2018

Notification	Answer categories	n	%	95% CI
Upon suspicion of LD, an immediate report to the MMD or NIPH should be done immediately. Do you have a routine for who does this and how?	Yes	31	66	51;79
	No	10	21	11;36
	Don’t know	6	13	5;26
What is your routine (*n* = 37)	We report to the MMD where the patient lives	9	24	12;41
	We report to NIPH directly and do not contact the MMD	0	0	0;9
	We report to both the MMD and NIPH	13	35	20;53
	We notify to MSIS	4	11	3;25
	Don’t know	5	14	5;29
	Other (free text)	6	16	6;32
Do you find it easy to report the MMD or NIPH about a new case of LD?	Yes	23	49	34;64
	No	3	6	1;18
	Don’t know	21	45	30;60
Who notifies cases of LD to MSIS (submits the form)? Choose all that apply.	The responsible doctor	41	87	74;95
	The laboratory	18	38	25;54
	Don’t know	2	4	1;15
	Other (free text)	5	11	4;23
NIPH has published MSIS notification criteria for LD. Do you find these criteria clear?	Yes	31	66	51;79
	No	0		
	Don’t know	16	34	21;49
Use of MSIS data				
Do you find LD incidence data from MSIS useful?	Yes	39	83	69;92
	No	1	2	0;11
	Don’t know	7	15	6;28
Do you find LD incidence data from MSIS easy to access?	Yes	24	51	31;66
	No	7	15	6;28
	Don’t know	16	34	21;49
Which sources of data do you use to find incidence of LD? Choose all that apply	NIPH Infection Control Guidelines	12	26	14;40
	www.MSIS.no	24	51	36;66
	Annual reports from NIPH	6	13	5;26
	Don’t know	8	17	8;31
	Other, please specify (free text)	6	13	5;26

Answers (*n* = 47) regarding notification and immediate reporting upon suspicion of Legionnaires’ disease (LD) and use of MSIS data from a survey to doctors at Norwegian hospitals that had notified at least one case with LD in the period 2013 to 2017

## Discussion

We evaluated the national surveillance of LD through MSIS with regard to outbreak and case detection and capacity to detect changes in incidence by time, place, and person. Our results suggest that the system overall functions well but with some room for improvement. The results from the linkage of MSIS to another data source with hospital treated LD cases suggest possible under-reporting to MSIS, and that the sensitivity of the system can improve. The survey results suggested differences in representativeness and acceptability of the system, which supports that MSIS does not capture all cases of LD. For cases notified to MSIS, the timeliness and the data quality were good for key variables for the response. If under-reporting is consistent with regard to time, place and person, the system would allow for changes in incidence to be detected.

The estimated external completeness reflects that not all cases are notified to MSIS and smaller outbreaks may not be reported. However, the system has proven to be able to detect smaller LD outbreaks, for example one with five cases in 2008 (8). However, the under-reporting to MSIS suggested by our results may be overestimated. The NPR is an administrative register, and it is possible that some of patients found only in NPR had a tentative LD diagnosis that was later rejected. In addition, we found evidence of incorrectly recorded LD diagnoses in patients only found in NPR. The correctness, as well as the completeness, of diagnostic coding in NPR varies between diagnoses (26–28), and limitations were reported also for infectious diseases in NPR (29, 30). In addition, it is also possible that some patients found only in NPR fell ill and sought medical care abroad and were only admitted to the hospital in Norway for follow-up care. These groups of patients are not notified in MSIS, meaning the external completeness of MSIS is likely higher than estimated here. Further, one assumption of the capture-recapture method is that the two data sources are independent. However, both cases notified in MSIS and recorded in NPR were treated in the same hospital, and the assumption does not hold. The estimate of total number of cases is an over-estimate, which adds to a false low external completeness estimate for the system. One alternative to explore, in order to facilitate surveillance, may be real-time data linkage of NPR and MSIS.

Patients only found in MSIS reduce the external validity of MSIS. None of these patients with a Norwegian personal identification number could be manually matched to patients only in NPR. Some of them might be recorded with other diagnoses in NPR, for example pneumonia. It was beyond the scope of this study to investigate the LD diagnosis of patients found only in NPR or MSIS in more depth through a review of medical journals or full data from NPR including every diagnosis in the study period, and this would be interesting to assess further.

The internal completeness and internal validity were high for key variables, meaning the data quality was high for cases that were notified to MSIS. These attributes are important to be able to produce accurate statistics for different subgroups of patients, and the long-term effect of any interventions. Validating patient data against the population registry and contacting clinician and lab directly about empty data fields are routines which contribute to good data quality. Overall, the timeliness estimates for notified cases were fair. Because the date for immediate reporting is not always recorded in MSIS, but the date of notification from the clinician and laboratory is always included, the true timeliness of the surveillance is likely better than our estimates suggest.

Our survey suggested that both the representativeness (who is tested for LD and how) and acceptability (knowledge of notification criteria, routines for notification, use of MSIS data), were fair, but that the system is not used to its full potential. Improved representativeness and acceptability would increase the sensitivity of the system. Because the MSIS system is common to all notifiable diseases, a lack of awareness of notification criteria could potentially affect the surveillance of other conditions that are notifiable to MSIS. Several survey responses suggested lack of awareness of the “immediate reporting” component of the surveillance, which needs to be prompt to prevent further cases. The survey results also indicated a lack of standardised procedures for *Legionella* testing in hospitals. If case-ascertainment varies between hospitals and hospital units, this reduces the representativeness, as well as the sensitivity of the surveillance. Case-ascertainment was not explicitly part of our evaluation and we would need another study design to be able to assess this in depth.

Globally, it is assumed that LD is under-diagnosed and that *Legionella* is an under-recognised cause of pneumonia (31–34) and it may be beneficial to carry out a multi-country evaluation to identify common obstacles for surveillance. At this time, it is currently not possible to directly compare LD incidence in Norway to other countries as the prevalence of *Legionella* sources and travel patterns may differ. However, possible reasons that may also be relevant to Norway include that LD is considered a severe disease and patients with less severe illness may not be tested for *Legionella.* This is supported by our finding that standardised criteria for whom to test is lacking. Further, the commonly used UAG only detects *L. pneumophila* serogroup 1. Our survey suggested another test methodology is commonly applied if LD is suspected, but if treatment which covers also *Legionella* is initiated, and the patient recovers, the cause of the pneumonia may never be identified. This was mentioned in the free text comments of the survey (data not shown). In the US, surveillance was biased towards more severe LD cases, who were more likely to be tested for LD, missing those empirically treated with antibiotics active against *Legionella spp*. and/or not requiring hospitalization (35). Moreover, patients with travel-history were more likely to be tested for LD in the US (16), which our survey results also suggested. The internal completeness for the variables that define a case as travel-associated was in our evaluation good. For gastro-intestinal infections, the data quality of these variables was questionable, as completion depends on what the General Practitioner (GP) knows or assumes (36). The majority of LD cases diagnosed in Norway are reported as associated with (international) travel, and it is possible that the proportion travel-associated cases is over-estimated, if illness is assumed to be associated with any recent travel. However, if this increases the test activity for legionella, it will improve the sensitivity of the surveillance.

As expected, the UAG was the most frequently used diagnostic test for *Legionella*. The test sensitivity has limitations (10), meaning that although the survey suggested that another test method is commonly applied upon a negative UAG, it is possible that some cases caused by both *L. pneumophila* serogroup 1 and other serogroups or species go undiagnosed, which reduces surveillance system sensitivity. Moreover, not more than 64% stated that positive UAG results are confirmed with culture and isolation “always” or “usually”. The MSIS notification criteria do not require a confirmatory test to be carried out. In order to ensure the representativeness of the system, use of common (national) guidelines on both which patients to test for *Legionella*, and on confirmation of positive as well as negative results would be ideal. There are national guidelines for treatment of LD which also include diagnostic procedures (37) but those who in the survey stated that they do have routines referred to internal guidelines.

Before the study, one of our hypotheses was under-reporting to MSIS due to UAG carried out in the hospital units. Since the hospitals’ microbiological laboratories report all positive test results on any notifiable disease daily, one could assume that notification rates would benefit from tests carried out by the laboratories. However, because a high proportion of survey respondents stated the UAG analysis is carried out by the hospital laboratory, this under-reporting is likely not extensive. However, in this study we did not assess the completeness, timeliness, and routines for MSIS notification by the primary microbiological laboratories.

The demographics of notified cases were consistent with what one would expect for LD based on known risk factors, and so was the reported county, meaning no demographic group appeared over- or under-represented.

### Limitations of the evaluation

We cannot know to what extent the survey answers are representative of medical doctors (and other health care staff) in Norwegian hospitals in general. The roles and responsibilities varied between the respondents. We did not have access to names or contact details to individual doctors, and there was no way we would be able to reach every eligible doctor. We also did not receive responses from every hospital, or from every unit in each hospital that we wanted to reach. Nevertheless, the overall response rate was better than anticipated. The survey had six replies from doctors in units that would not be expected to treat LD patients, such as a cancer or women’s health clinic. We suspect that they might belong to a larger unit that we asked the survey to be forwarded to, such as general medicine units. However, the answers from such units did not stand out as having many “don’t know” answer and were retained in the data.

Although the clinical criteria for notification of LD to MSIS is pneumonia, it is theoretically possible that a patient with Pontiac fever, a milder non-pneumonic form of legionellosis, could be notified to MSIS. However, such patients would in Norway visit their GP who is highly unlikely to request a test for legionellosis. For the linkage of MSIS and NPR we assumed any patients with LD would be treated in a hospital, not by a GP whose reimbursement claims are not in NPR.

## Conclusions and recommendations

Our results suggest that the national LD surveillance in MSIS can detect changes in incidence of LD over time, and by place and person, but likely does not detect every case of LD diagnosed in Norway, which could weaken its ability to detect outbreaks. Although our survey results cannot be regarded as representative for all Norwegian hospital doctors, we found indications of sub-optimal representativeness mainly because of high variability in hospital diagnostic procedures. We recommend further investigation of when patients are tested for LD, what diagnostic tests are used, and routines for confirmation of positive as well as negative UAG results. The survey further suggested sub-optimal acceptability of the surveillance. We recommend a more in depth assessment of hospital doctors of awareness of and experience from applying the MSIS notification criteria and immediate reporting upon clinical suspicion of LD. We also recommend a more comprehensive assessment of the patients only registered in NPR, to learn whether these patients were diagnosed with LD. Finally, we recommend an assessment of the laboratories’ notification routines as they should also notify LD to MSIS, and they were not part of this evaluation.

## Supplementary information

**Additional file 1.** Survey questionnaire, English translation of the questions (originally in Norwegian)

## Data Availability

The data that support the findings of this study are available from MSIS and the Norwegian Patient Registry (NPR) but restrictions apply to the availability of these data, which were used after approval for the current study, and so are not publicly available. Data from MSIS are however available from the authors upon reasonable request and with permission of MSIS (www.fhi.no/hn/helseregistre-og-registre/msis). Data from the Norwegian Patient Registry have been used in this publication. The interpretation and reporting of these data are the sole responsibility of the authors, and no endorsement by the Norwegian Patient Registry is intended nor should be inferred.

## Abbreviations

BAL: Bronchoalveolar lavage
CDC: The U.S. Centers for Disease Control and Prevention
EC: External completeness
ECDC: European Centre for Disease Prevention and Control
ELDsnet: European Legionnaires´ Disease Surveillance Network
GP: General practitioner
HDir: Norwegian Directorate of Health
LD: Legionnaires´ disease
MMD: Municipality medical doctor
MSIS: The Norwegian surveillance system for communicable diseases
NIPH: Norwegian Institute of Public Health
NPR: Norwegian Patient Registry
PPV: Positive predictive value
UAG: Urine antigen test
