# Supplementary material for: Evaluation of the national surveillance of Legionnaires' disease in Norway, 2008-2017
Source: BMC Public Health. 2019 Dec 3;19:1624. doi: 10.1186/s12889-019-7981-9 (PMC6889696; doi:10.1186/s12889-019-7981-9)
Supplement: Supplementary file 1 — Additional file 1. Survey questionnaire, English translation of the questions (originally in Norwegian) [file 12889_2019_7981_MOESM1_ESM.docx]

# Additional file 1: English translation of questions

1. Please state which unit and which hospital you respond on behalf of

2. Please state the name, title and contact details (telephone number and email) of the person answering the survey in case we would need additional information

3. Does the hospital or hospital unit have an internal procedure or algorithm for which patients to test for *Legionella*?

- Yes, one that all doctors should follow
- No, it is up to each responsible doctor
- No, other
- Don’t know

4. If yes: Which internal procedure or algorithm, please give a brief description

5. Do you test all patients with suspected pneumonia for Legionella?

- Yes
- No
- Don’t know

6. Do you test all patients with suspected pneumonia and a travel history for Legionella?

- Yes
- No
- Don’t know

7. Which diagnostic test(s) do you use at your unit to diagnose/confirm Legionella? Choose all that apply

- Urine antigen test
- Culture and isolation
- PCR
- Serology
- Don’t know
- Other (free text)

If urine antigen test is used: 8. Where is the urine antigen test normally carried out?

- At the hospital unit
- By the hospital laboratory
- It varies, both at the unit and by the laboratory
- Don’t know
- Other (free text)

If urine antigen test is used: 9. Do you try to confirm a positive urine antigen test with culture and isolation of BAL or sputum?

- Yes, always
- Yes, usually
- Rarely
- No, never
- Don’t know

If urine antigen test is used: 10. What is your routine if the clinical suspicion is LD but the urine antigen test is negative?

- Sample for analysis with another method (culture, PCR, serology)
- Don’t know
- Other (free text)
- Yes, always

If PCR is used: 11. Do you try to confirm a positive PCR result with culture and isolation of BAL or sputum?

- Yes, usually
- Rarely
- No, never
- Don’t know

If serology is used: 12. Do you try to confirm a positive serology result with another diagnostic method?

- Yes, always
- Yes, usually
- Rarely
- No, never
- Don’t know

If serology is used: 13. Is a positive serology result confirmed with a new sample with regard to titre increase?

- Yes, always
- Yes, usually
- Rarely
- No, never
- Don’t know

14. Upon suspicion of LD, an immediate report to the MMD or NIPH should be done immediately. Do you have a routine for who does this and how?

- Yes
- No
- Don’t know

If yes or don’t know: 15: What is your routine?

- We report to the MMD where the patient lives
- We report to NIPH directly and do not contact the MMD
- We report to both the MMD and NIPH
- We notify to MSIS
- Don’t know
- Other (free text)

16. Do you find it easy to report the MMD or NIPH about a new case of LD?

- Yes
- No
- Don’t know

If no: 17. Why is it not easy to report?

(free text)

18. Who notifies cases of LD to MSIS (submits the form)? Choose all that apply.

- The responsible doctor
- The laboratory
- Don’t know
- Other (free text)

19. NIPH has published MSIS notification criteria for LD. Do you find these criteria clear?

- Yes
- No
- Don’t know

If No: 20: Why are the criteria not clear?

(free text)

21. Do you find LD incidence data from MSIS useful?

- Yes
- No
- Don’t know

22. Do you find LD incidence data from MSIS easy to access?

- Yes
- No
- Don’t know

23. Which sources of data do you use to find incidence of LD? Choose all that apply

- NIPH Infection Control Guidelines
- www.MSIS.no
- Annual reports from NIPH
- Don’t know
- Other, please specify (free text)

24, Do you have any other comments on surveillance of LD?

(free text)
